# Construction and verification of a novel prognostic risk model for kidney renal clear cell carcinoma based on immunity-related genes

**DOI:** 10.3389/fgene.2023.1107294

**Published:** 2023-01-20

**Authors:** Yufeng Liu, Dali Wu, Haiping Chen, Lingfei Yan, Qi Xiang, Qing Li, Tao Wang

**Affiliations:** Department of Urology, The Fifth Affiliated Hospital, Southern Medical University, Guangzhou, China

**Keywords:** kidney renal clear cell carcinoma, risk model, prognostic biomarker, immune infiltration, KIRC

## Abstract

**Background:** Currently, there are no useful biomarkers or prognostic risk markers for the diagnosis of kidney renal clear cell carcinoma (KIRC), although recent research has shown that both, the onset and progression of KIRC, are substantially influenced by immune-associated genes (IAGs).

**Objective:** This work aims to create and verify the prognostic value of an immune risk score signature (IRSS) based on IAGs for KIRC using bioinformatics and public databases.

**Methods:** Differentially expressed genes (DEGs) related to the immune systems (IAGs) in KIRC tissues were identified from The Cancer Genome Atlas (TCGA) databases. The DEGs between the tumor and normal tissues were identified using gene ontology (GO) and Kyoto Encyclopaedia of Genes and Genomes (KEGG) enrichment analyses. Furthermore, a prognostic IRSS model was constructed and its prognostic and predictive performance was analyzed using survival analyses and nomograms. Kidney renal papillary cell carcinoma (KIRP) sets were utilized to further validate this model.

**Results:** Six independent immunity-related genes (PAEP, PI3, SAA2, SAA1, IL20RB, and IFI30) correlated with prognosis were identified and used to construct an IRSS model. According to the Kaplan-Meier curve, patients in the high-risk group had significantly poorer prognoses than those of patients in the low-risk group in both, the verification set (*p* <0.049; HR = 1.84; 95% CI = 1.02–3.32) and the training set (*p* < 0.001; HR = 3.12, 95% CI = 2.23–4.37). The numbers of regulatory T cells (Tregs) were significantly positively correlated with the six immunity-related genes identified, with correlation coefficients were 0.385, 0.415, 0.399, 0.451, 0.485, and 0.333, respectively (*p* <0.001).

**Conclusion:** This work investigated the association between immune infiltration, immunity-related gene expression, and severity of KIRC to construct and verify a prognostic risk model for KIRC and KIRP.

## Introduction

Renal cell carcinomas (RCCs) are malignancies in the kidneys; globally, the incidence of RCC has been steadily rising over the last few decades. The most common types of RCCs are kidney renal clear cell carcinomas (KIRCs) and kidney renal papillary cell carcinomas (KIRPs). Although in the early stages of both diseases, patients often display no clinical symptoms, the prognosis for those with KIRP is better than for those with KIRC. It is also unclear if KIRC and KIRP have similar pathogenesis pathways.

KIRC represents about 80% of all RCC cases in adults, whereas KIRP about 10%–15% (Ricketts et al., 2018). For most cases of localized KIRC, the primary treatment consists of surgical removal of the tumor; for advanced and metastatic cases, however, chemotherapy, targeted medication, and immunotherapy are usually used (Chen et al., 2019). When diagnosed at an advanced stage, RCC is a fatal illness with a dismal 5-year survival rate of 11.7% (Siegel et al., 2017). Currently, combinations of anti-VEGF (Vascular Endothelial Growth Factor, VEGF) and immune checkpoint inhibitor treatments are under investigation, which if successful, will significantly alter the therapeutic landscape for RCC treatment (Motzer et al., 2015).

Since immune-associated genes (IAGs) are known to significantly influence the onset and progression of KIRC, (Ghatalia et al., 2019; Xu et al., 2019), several types of immunotherapies, including the use of programmed death-1 (PD-1) inhibitors, cytotoxic T-lymphocyte associated protein 4 (CTLA-4) inhibitors, or programmed death-ligand 1 (PD-L1) inhibitors, are proving to be effective treatment options (Atkins and Tannir, 2018). Recently, a multicentre, randomized, double-blind, placebo-controlled, phase 3 trial has shown that Pembrolizumab is an effective immunotherapeutic agent for KIRC (Powles et al., 2022). However, poor long-term response rates and the absence of efficient prognostic predictors have restricted the use of such first-generation immunotherapies. Therefore, there is an urgent need for identifying new biomarkers, especially immune-related ones, to track disease development, prognosis, and therapy response in KIRC patients.

In recent years, many studies have reported on how prognostic models can be valuable clinical tools in the treatment of various conditions (Huang et al., 2019; Shen et al., 2019; Xu et al., 2020a; Liu et al., 2020; Qu et al., 2020; Wang et al., 2020; Jia et al., 2022; Zhang et al., 2022). However, there have been few investigations on establishing prognostic models for KIRC, especially those related to the immune system. To investigate the relationships between the immune system and the progression of KIRC, we used data from The Cancer Genome Atlas (TCGA) database to construct a risk score model relying on immunity-related genes. Collectively, our results demonstrate the functional significance of immunity-related signatures as prognostic biomarkers for KIRC and kidney renal papillary cell carcinoma (KIRP).

## Materials and methods

### Data compilation and handling

We retrieved data on gene expression, clinicopathology, and survival of patients with KIRC and KIRP from The Cancer Genome Atlas (TGCA) database and the University of California Santa Cruz (UCSC)database. The KIRC samples with prognostic data, comprising 172 normal samples and 539 tumor samples, were integrated to form a training set. A verification set containing prognostic data on 256 KIRP patients was also established.

In total, the expression profiles of 2,483 immunity-related genes were retrieved, with the complete gene names obtained from the Immunology Database and Analysis Portal (ImmPort) database’s Gene List module (Supplementary Table S1).

### The human protein atlas (HPA) databases

The HPA contains extensive data on the transcriptome and proteome of various human specimens, including tissue, cell, and pathology atlases. Moreover, the database also provides data on protein immunohistochemistry in tumors and normal human tissue samples.

### Differential gene expression analysis

Differential gene expression analysis was used to identify the differentially expressed genes (DEGs) between the tumor and normal groups. We used the ‘limma’ software package in R (v. 3.6.3), With a false discovery rate (FDR) < 0.25 and Log2| Fold Change | > 2. The DEGs were visualized through volcano plots using the “ggplot2,” “Cairo,” and “ggrepel” packages in R (v. 3.6.3).

### Gene ontology (GO) and kyoto encyclopedia of genes and genomes (KEGG) analysis

We used the ‘clusterProfiler’ package (v. 3.14.3) to perform GO and KEGG analyses to discover the prospective biological functions and enrichment pathways of the identified DEGs. The KEGG database incorporates information on chemical, genomic, and systematic functional data. The molecular functions, biological processes, and cell composition are gene features that are primarily described by the GO analysis. An adjusted *p*-value of <0.05 indicated significance. We used the “ggplot2” package (version 3.3.3) for visualization.

### Establishment of immune risk score signature (IRSS) for prognosis

To identify the differentially expressed immunity-related genes (DEIGs), a total of 1734 genes associated with immunity were identified in KIRC tissues and compared with the DEGs obtained in the previous analysis. Univariate Cox regression analysis was then used to identify immunity-related genes that had significant relationships with prognosis. Following this, we used LASSO (least absolute shrinkage and selection operator) regression analysis to identify a subset of genes that could be used as independent prognostic indicators. The LASSO regression allowed us to improve our model’s accuracy and interpretability, while also addressing the issue of collinearity among the independent variables (Alhamzawi and Ali, 2018). Independent prognostic factor-related regression coefficients were then obtained from multivariate Cox regression analysis. Lastly, the formula for an IRSS, which was established using the multivariate Cox regression coefficient beta value as:

IRSS=EXPgene1*β1+EXPgene2*β2+EXPgene3*β3+. . .+EXPgenen*βn

wherein this formula, EXP denotes the expression level of the gene and β signifies the regression coefficient from the multivariate Cox regression analysis (Zeng et al., 2017).

Groups with high-IRSS and low-IRSS were established where the risk score for each TCGA-KIRC sample was computed using the above formula; the median risk score was used as the cut-off value for the establishment of these groups. Additionally, the log-rank test was used for comparing overall survival (OS) rates between the two groups, which was visualized using Kaplan-Meier (KM) curves. The AUC (area under the curve) for the receiver operating characteristic (ROC) was used to analyze the prognostic value of the six immunity-related genes identified in this study and the IRSS in KIRC patients. The horizontal and vertical axes of the ROC curve denote specificity and sensitivity, respectively (DeLong et al., 1988). The AUC provides a probability value that can be used to assess the model’s predictive accuracy and varies in value from 0.5 to 1, where the larger the value, the better the model’s accuracy. In our study, larger AUC values denote higher levels of agreement between the actual OS and the predicted OS. For these analyses, we used the “glmnet” package (version 4.1-2) and the ‘survival’ package (version 3.2-10).

### Immune cell infiltration analysis

Marker genes for each of the 24 types of immune cells were obtained from a research report by Bindea et al. (2013). We used the single sample Gene Set Enrichment Analysis (ssGSEA) method to identify the different immune cells that infiltrated KIRC tumors. We used Wilcoxon’s signed-rank sum test and Spearman’s correlation to analyze the associations between the gene expression profiles of the six DIEGs and immune infiltration patterns. The analysis was performed using the “Xiantao tool” module (https://www.xiantao.love/products) and the ‘GSVA’ package (version 1.34.0).

### Analyzing the relationships between clinicopathological data and the IRSS

We screened for KIRC-related prognostic predictors, specifically clinical characteristics, and examined their association with the IRSS that we have developed. We paid special attention to the relationship between the OS and the IRSS, which was examined with the univariate Cox proportional risk model. Multivariate Cox regression analysis was used to establish the potential of the IRSS as an independent prognostic predictor. A nomogram was constructed by incorporating clinicopathological data from KIRC patients, including age, gender, histologic grade of the tumor, pathologic stage of the tumor, and IRSS; this was used to comprehensively evaluate patients' survival data. We used the “rms” package [version 6.2-0] and the ‘survival’ package [version 3.2-10] for these analyses.

In addition, decision curve analysis (DCA) were also generated across 1, 3, and 5 years to assess the suitability of nomograms for clinical use. The threshold probability percentage and net income are displayed in a graph (Figure 8) on the *x* and *y*-axes, respectively.

### Verification of the IRSS

The TCGA-KIRP dataset (*n* = 256) was chosen to verify our IRSS model and assess the generalization of the signature since KIRC and KIRP tumors are anatomically and histologically quite similar. Every patient’s risk score in the KIRP cohort was computed and ranked using the IRSS formula that we developed using the TCGA-KIRC dataset. The TCGA-KIRP cohort’s cutoff values were used to classify the KIRP samples into groups with high- and low-risk as per the scores. The KM curve was used to compare survival across the two groups. The ROC curve was used to evaluate the precision of the signature prediction. In addition, the nomogram was used to evaluate the survival probabilities of the KIRP patients using clinicopathological data, including age, gender, histologic grade of the tumor, pathologic stage of the tumor, and the IRSS.

## Results

### Differential gene expression analysis

**FIGURE 1:**
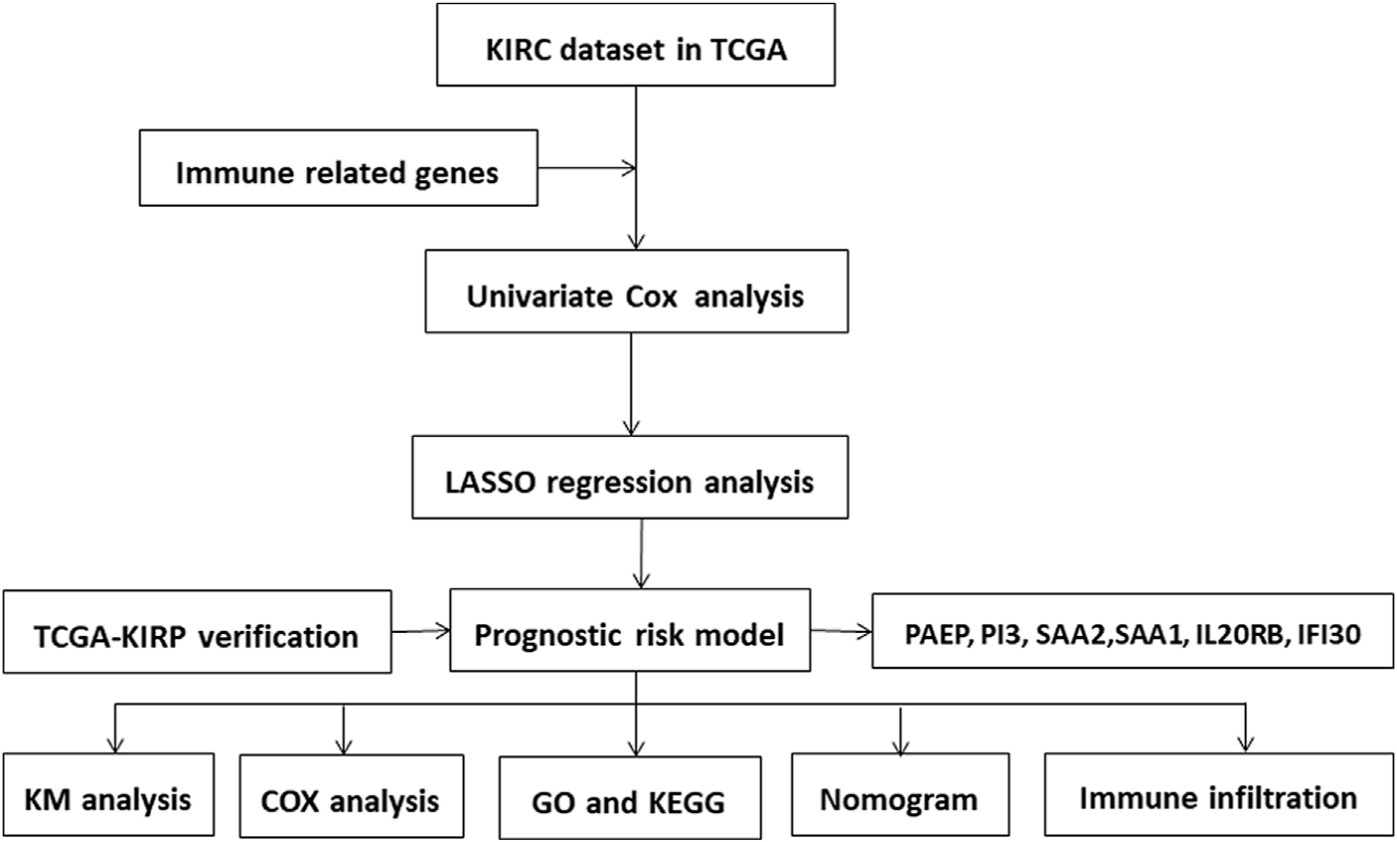
Flow chart of steps followed for data collection and analysis in this study.

**FIGURE 2:**
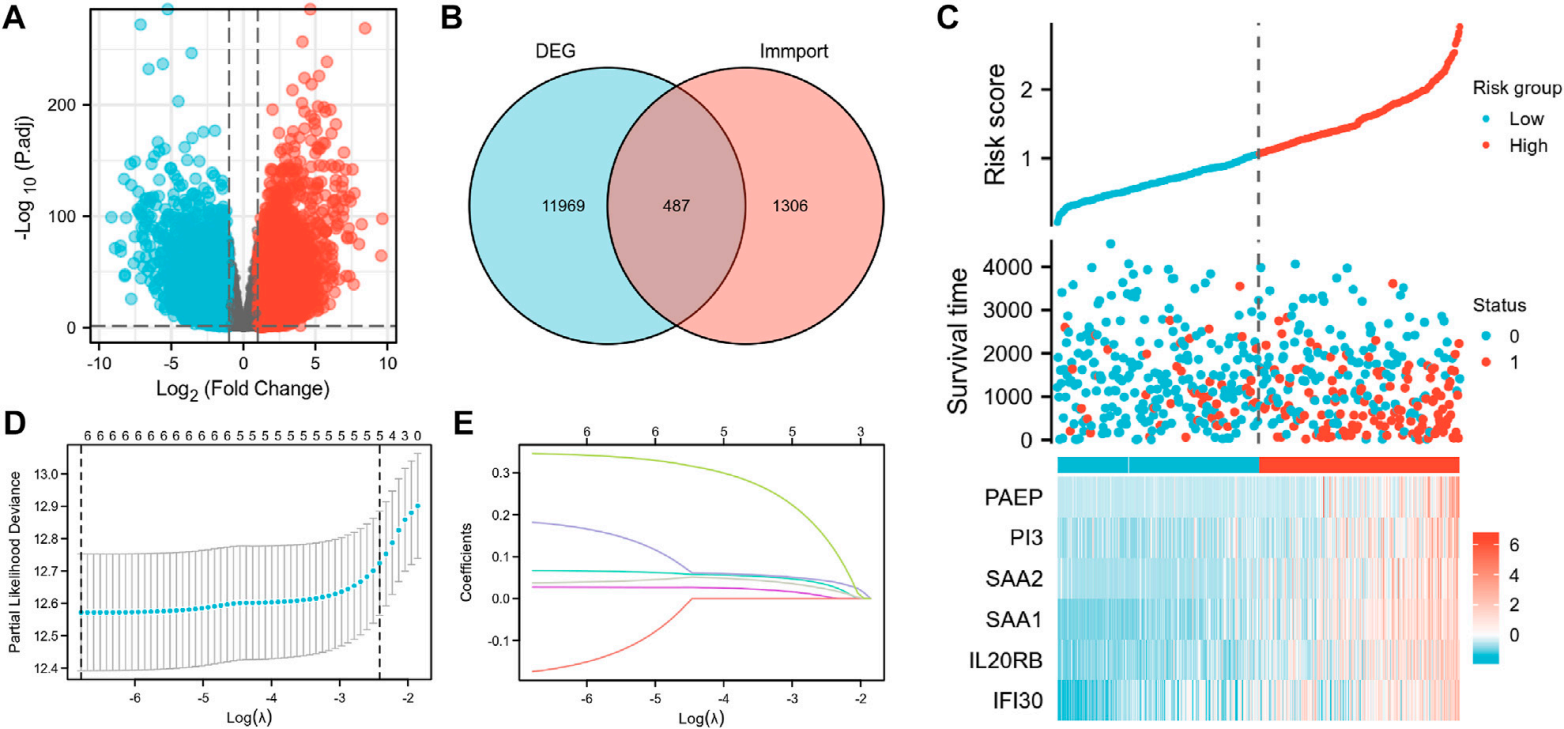
Construction of the IRSS. **(A)** Volcano plot of differentially expressed genes. **(B)** Intersection Venn diagram of DEGs and immunity-related genes. **(C)** Risk scores, survival status, and heat maps of six immunity-related genes in KIRC patients. **(D)** Ten-fold cross-validation of the LASSO model-related optimized parameter selection. **(E)** LASSO coefficient profiles. 0 represents survival status “alive,” one represents survival status “dead”.

We first created a flow chart (Figure 1) to illustrate the steps followed in this study. The training set had samples from 539 KIRC patients along with 172 normal samples with data on gene expression, prognosis, and survival. A risk score model was developed based on the expression patterns of the immunity-related genes to predict the prognoses of the KIRC patients to examine if these genes can be used as biomarkers. The differential gene expression analysis identified 6,533 DEGs (|log2(FC)|>2 and *p* <0.05) as depicted in the volcano plot (Figure 2A) Supplementary Table S2. Of these, 5,055 were upregulated (logFC positive) and 1,478 were downregulated (logFC negative).

### GO and KEGG analysis

**FIGURE 3:**
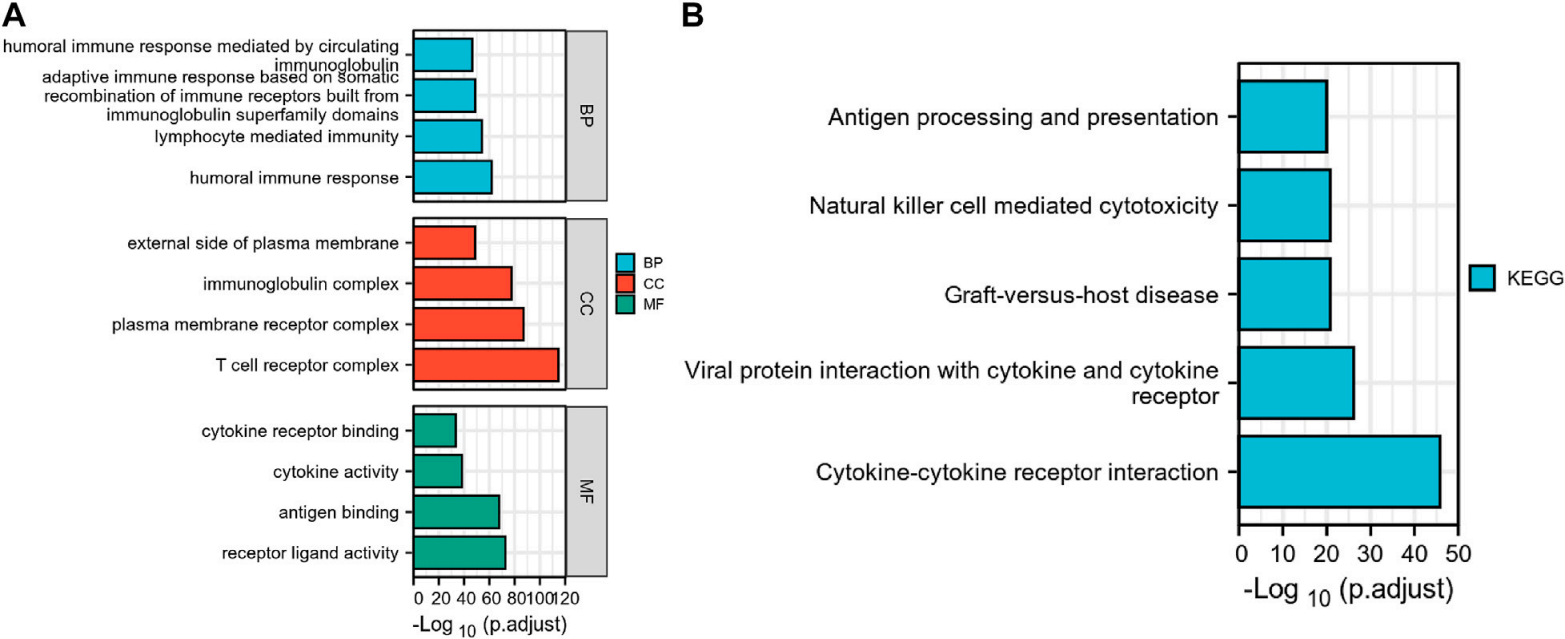
Differential genes expressed in normal and KIRC tissues are involved in immune-related pathways. **(A)** GO, Gene Ontology; **(B)** KEGG, Kyoto Encyclopedia of Genes and Genomes.

GO and KEGG enrichment analyses were conducted to investigate the prospective relationship between immune activity and gene expression in the tumor and normal tissues in the TCGA-KIRC cohort. The DEGs were enriched in a range of processes in both tissue types, with immune-related pathways making up the majority of these processes. As per the KEGG enrichment analysis (Figure 3), natural killer cell-mediated cytotoxicity, cytokine-cytokine receptor interaction, BP modules implicated in receptor-ligand activity, cytokine receptor binding, and cytokine activity, in GO enrichment analysis. Collectively, these results demonstrate that certain immune genes are differentially expressed in KIRC tumors. Supplementary Table S3, S4 provide more details on the enrichment analysis results.

### Construction and prognostic value of IRSS

According to the Venn diagram showing the overlay of the immunity-related genes and DEGs (Figure 2B); 487 DEIGs were identified (Supplementary Table S5; Figure 2B). Of these, six genes that had a strong link to prognosis were found during the LASSO regression analysis. Merging the results depicted in Figures 2D, E, the six immunity-related genes that were selected and included in the model were PAEP, PI3, SAA2, SAA1, IL20RB, and IFI30 (Figure 2C; Supplementary Table S6). The associated regression coefficients for these genes (β1–β6) were 0.067, 0.027, −0.174, 0.182, 0.038, and 0.346, respectively (Supplementary Table S7). The IRSS was developed using the following formula:

IRSS=EXP PAEP*0.067+EXP PI3 *0.027+EXP SAA2 *−0.174+EXP SAA1*0.182+EXP IL20RB * 0.038+EXP IFI30 *0.346

**FIGURE 4:**
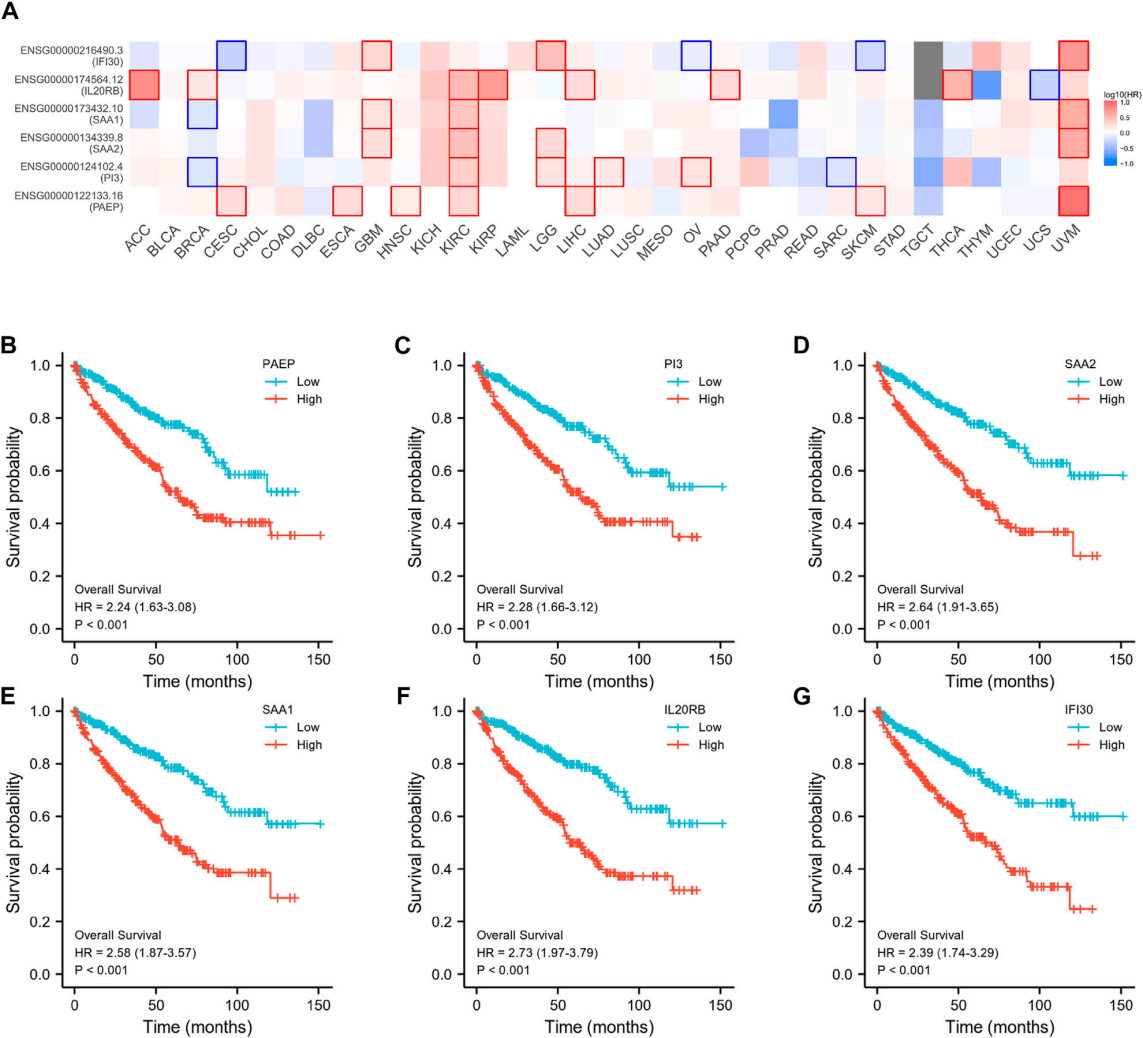
Prognostic analysis of six immunity-related genes in KIRC. **(A)** Establishment of an OS heatmap of these genes. **(B–G)** Survival analysis to investigate the association between the expression levels of PAEP, PI3, SAA2, SAA1, IL20RB, IFI30, and overall survival of KIRC.

**FIGURE 5:**
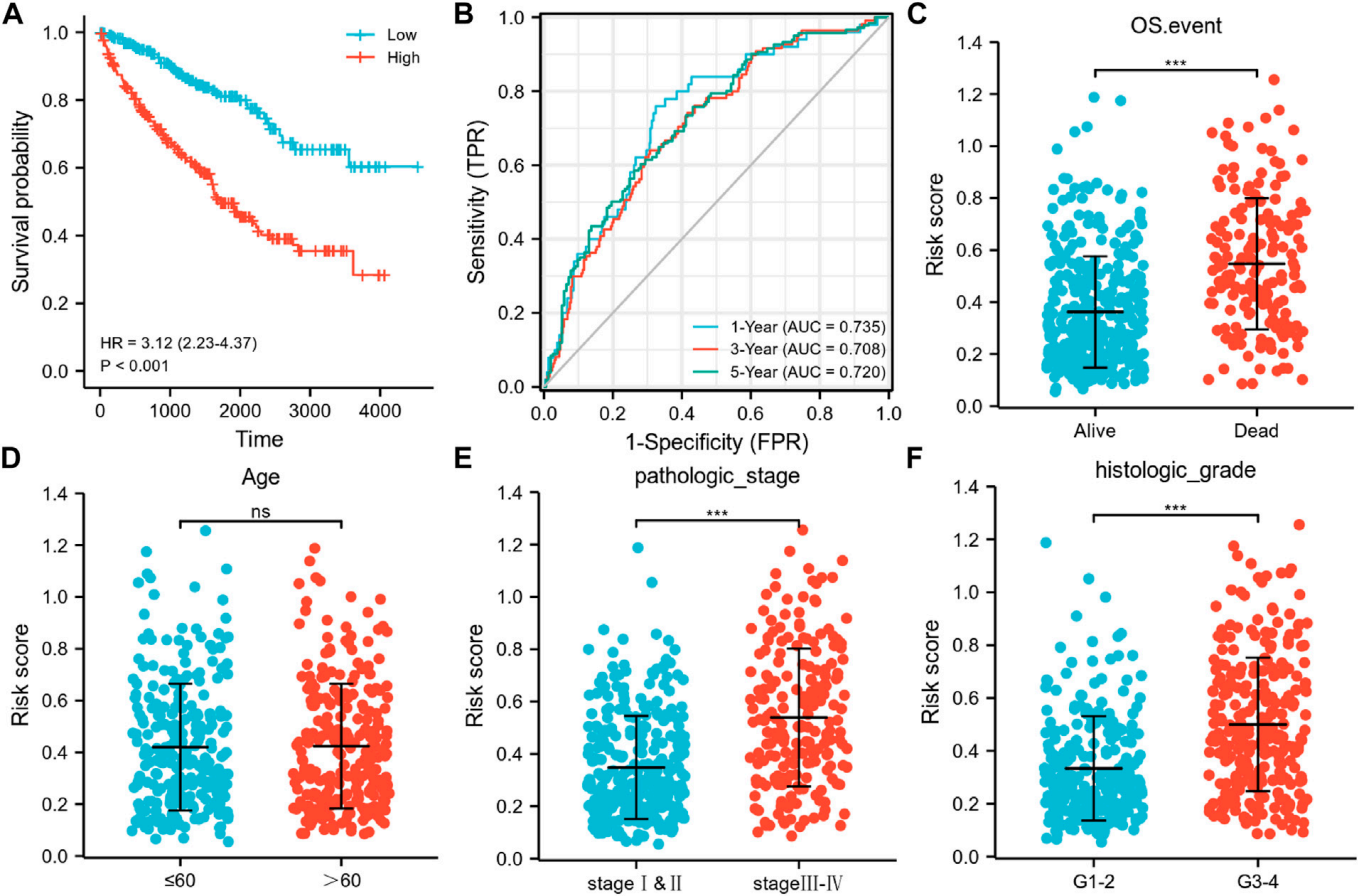
Associations of the six immunity-related genes signatures with OS, AUC curves, and clinicopathological features in KIRC. **(A)** Kaplan-Meier curve exhibited substantial variation in OS across the high- and low-risk groups in the TCGA-KIRC cohort. **(B)** Time-dependent ROC curves for predicting 1-, 3-, and 5-year survival rates. Relationship between signature risk scores and clinicopathological features in **(C)** OS events (Alive vs. Dead; unpaired Student’s t-test, *p* <0.001), **(D)** age (>60 vs. ≤60; unpaired Student’s t-test, *p* >0.05), **(E)** Pathological stage of tumor (stage I–II vs stage III–IV; unpaired Student’s t-test, *p* <0.001), **(F)** Histological grade of tumor (G1–2 vs G3–4; unpaired Student’s t-test, *p* <0.001).

**FIGURE 6:**
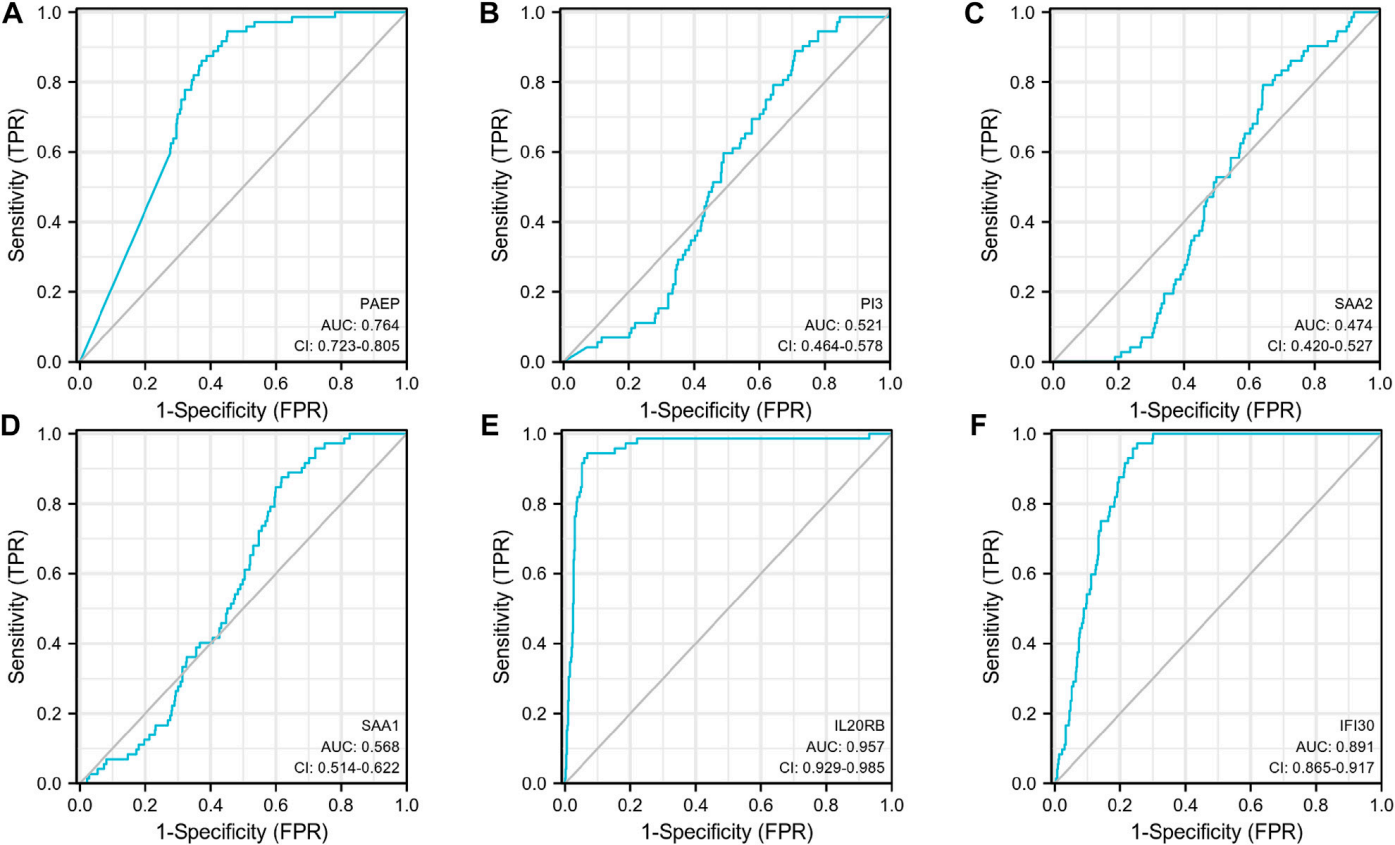
Single-indicator ROC curves for PAEP, PI3, SAA2, SAA1, IL20RB, and IFI30 for diagnosis of KIRC. **(A)** PAEP, **(B)** PI3, **(C)** SAA2, **(D)** SAA1, **(E)** IL20RB, **(F)** IFI30.

Subsequently, an OS heatmap of the six immune-related genes was generated (Figure 4A) and the relationship between the IRSS and prognosis in KIRC patients was examined using the Kaplan-Meier (K-M) plotter. As shown in Figures 4B–G, high expression levels of PAEP, PI3, SAA2, SAA1, IL20RB, and IFI30 is correlated with poor prognosis in patients with KIRC. We also analyzed the relationships between the expression levels of the six immunity-related genes and overall survival outcomes in KIRP. The results showed that high expression levels of SAA2, SAA1, and IL20RB are linked to poor prognosis (Supplementary Figure S1). Additionally, when the KIRC samples were split into low- and high-risk groups based on the IRSS, we found that patients in the high-risk group had poorer prognoses than those in the low-risk group (Figure 5A, log-rank *p* < 0.001; HR = 3.12, 95% CI = 2.23–4.37). Subsequently, the single-indicator ROC curve analysis indicated that AUC (Area under curve) values were 0.764, 0.521, 0.474, 0.568, 0.957, and 0.891, respectively. (Figure 6). Following this, the effectiveness of our model in predicting OS for KIRC patients was evaluated using the ROC curves. As shown in Figure 5B, the 1-, 3-, and 5-year AUC values were 0.735, 0.708, and 0.720, respectively, which demonstrates the model’s reliability and accuracy in predicting patient prognosis.

### Associations between the IRSS and the clinicopathological characteristics of KIRC

An investigation of the relationships between the clinicopathological characteristics and the IRSS revealed that the progression of KIRC was closely associated with the six immunity-related genes identified in this study. Patients with pathological stage III–IV and histologic grade G three to four tumors, and those who died because of KIRC were found to have greater risk scores as compared to those with pathological stage I–II (*p* <0.001) and histologic grade G 1–2 (*p* <0.001) tumors and patients who survived (*p* <0.001; Figures 5C, E, F). No significant association between age and the IRSS were observed (*p* >0.05; Figure 5D). Overall, our findings demonstrated that the six immunity-related genes identified in this study are strongly linked to KIRC progression.

### Correlations between immune infiltration and expression levels of PAEP, PI3, SAA2, SAA1, IL20RB, and IFI30

**FIGURE 7:**
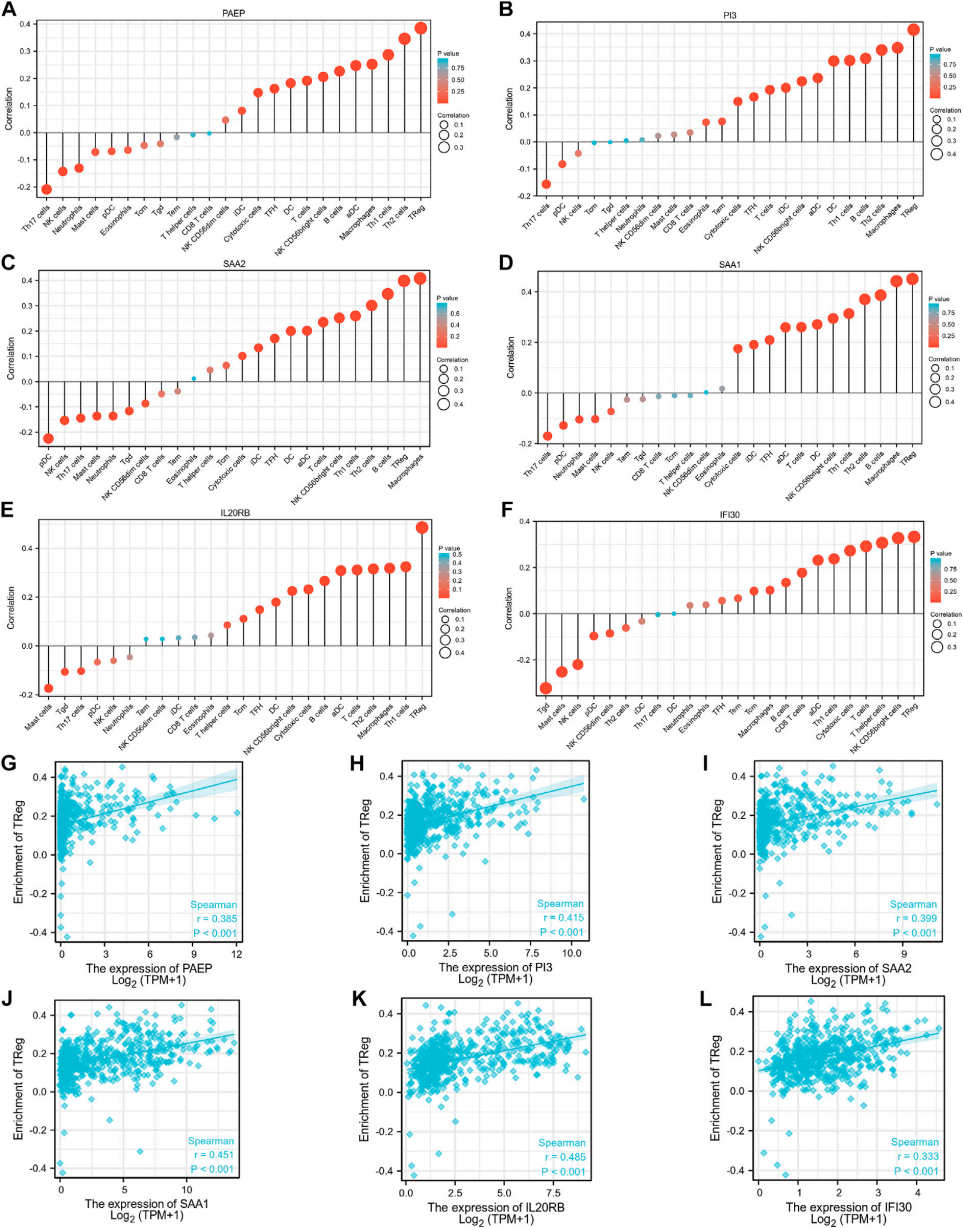
Associations between expression levels of the six immunity-related genes and infiltrating immune cells in patients with KIRC. **(A–F)** The associations between the expression levels of PAEP, PI3, SAA2, SAA1, IL20RB, and IFI30 and infiltration by immune cells. **(G–L)** The associations between expression levels of PAEP, PI3, SAA2, SAA1, IL20RB, IFI30 and infiltration by regulatory T cells in KIRC tissues.

As shown in Figure 7, PAEP expression was significantly positively correlated with infiltration by regulatory T cells (Tregs), Th2 cells, and Th1 cells (*p* <0.001); PI3 expression was significantly positively correlated with infiltration by Tregs, macrophages, and Th2 cells (*p* <0.001); SAA2 expression was significantly positively correlated with infiltration by macrophages, Tregs, and B cells (*p* <0.001); SAA1 expression was significantly positively correlated with infiltration by Tregs, macrophages, and B cells (*p* <0.001); IL20RB expression was significantly positively correlated with infiltration of Tregs, Th1 cells, and macrophages (*p* <0.001); IFI30 expression was significantly positively correlated with infiltration by Tregs, NK CD56 bright cells, and T helper cells (*p* <0.001). Interestingly, we found that Tregs were positively associated with all the six genes identified in this study, with correlation coefficients of 0.385, 0.415, 0.399, 0.451, 0.485, and 0.333, respectively. We also analyzed the relationships between the six immunity-related genes identified here and 24 tumor-infiltrating immune cells in KIRP tissues. The results showed that the six immunity-related genes were also associated with multiple immune cell infiltration in KIRP (Supplementary Figure S2).

### Prognostic significance of IRSS

**FIGURE 8:**
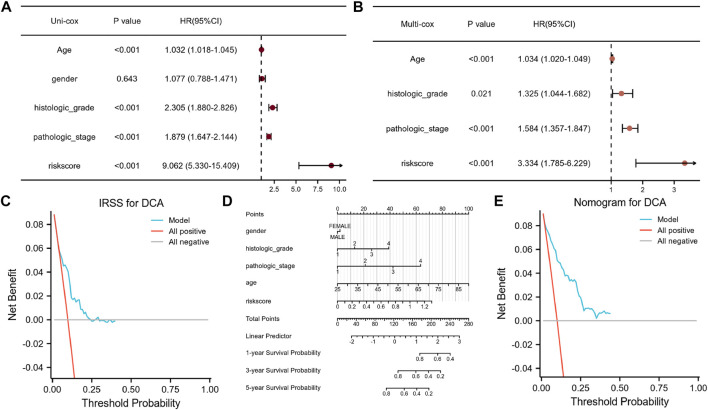
Assessment of the IRSS along with establishment and assessment of nomograms. **(A)** Univariate Cox regression analysis indicated that age, gender, histological grade of the tumor, pathological stage of the tumor, and risk score were correlated with OS in KIRC patients. **(B)** Multivariate Cox regression analysis indicated that the risk score was correlated with the OS in KIRC patients. **(C)** Decision curve analysis (DCA) for assessing the performance of the IRSS in prognostic prediction. **(D)** 1-, 3-, and 5-year nomograms for predicting OS in patients with KIRC. This nomogram incorporates the following five variables: Age, gender, histological grade of the tumor, pathological stage of the tumor, and IRSS. **(E)** Decision curve analysis (DCA) for the assessing the performance of the nomogram.

We used univariate and multivariate Cox regression analyses to evaluate the prognostic significance of the IRSS that we have developed. According to univariate Cox regression analysis, in the TGGA-KIRC dataset, age, histologic grade, pathologic stage, and risk score, were all prognostic predictors, although gender was not (Figure 8A). According to the multivariate Cox regression analysis (Figure 8B, log-rank *p* < 0.001; HR = 3.334, 95% CI = 1.785–6.229; Table 1), the risk score was an independent prognostic predictor. According to the DCA (Figure 8C), the IRSS in combination with various clinicopathological characteristics has greater clinical applicability. According to the aforementioned findings, our IRSS can be employed as a robust and innovative prognostic biomarker for KIRC.

**TABLE 1:** Univariate and multivariate Cox regression analysis.

Characteristics	Total(N)	Univariate analysis		Multivariate analysis	
		Hazard ratio (95% CI)	*p*-value	Hazard ratio (95% CI)	*p*-value
gender	526				
MALE	346	Reference			
FEMALE	180	1.077 (0.788–1.471)	0.643		
histologic_grade	526	2.305 (1.880–2.826)	**<0.001**	1.325 (1.044–1.682)	**0.021**
pathologic_stage	526	1.879 (1.647–2.144)	**<0.001**	1.584 (1.357–1.847)	**<0.001**
age	526	1.032 (1.018–1.045)	**<0.001**	1.034 (1.020–1.049)	**<0.001**
riskscore	526	9.062 (5.330–15.409)	**<0.001**	3.334 (1.785–6.229)	**<0.001**

Bolded font means statistically significant.

Nomograms are useful because they simplify statistical prediction models to single numerical estimates of event possibilities suited to specific patient characteristics (Iasonos et al., 2008). Since numerous clinical characteristics have prognostic significance in clinical practice, we constructed a nomogram using numerous clinicopathological characteristics and the IRSS to predict patient prognosis in KIRC. As depicted in Figure 8D, it is possible to compute and combine the scores of each variable to generate a prognosis. According to the DCA (Figure 8E), the nomogram’s clinical application value is also higher when in combination with a variety of clinical variables.

### Verification of IRSS using the TCGA-KIRP cohort

**FIGURE 9:**
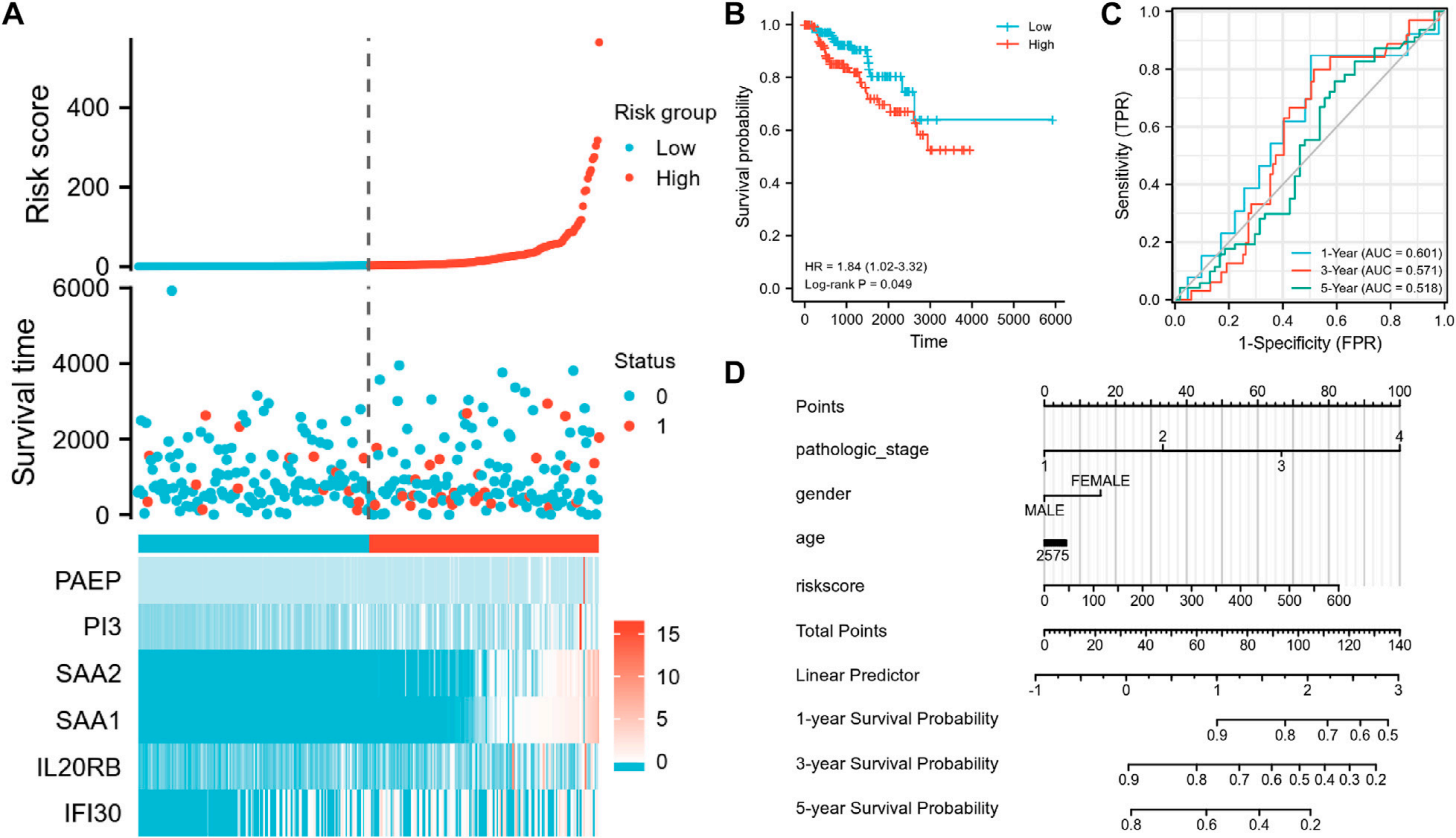
Validation of the IRSS signature with the TCGA-KIRP dataset. **(A)** Risk scores, survival status, and heat maps of the six immunity-related genes in KIRP patients. **(B)** Kaplan-Meier curve exhibited significant differences in OS between the high- and low-risk groups in TCGA-KIRP. **(C)** Time-dependent ROC curves for predicting 1-, 3- and 5-year survival rates. **(D)** 1-,3-, and 5-year nomograms for predicting OS in patients with KIRP. This nomogram incorporates the following four variables: Age, gender, pathology stage, and IRSS. 0 represents the survival status “alive” and one represents the survival status “dead.”

Data from the TCGA-KIRP cohort were used as a verification dataset to validate the usability of the IRSS that we have developed for RCCs. After the individual patients’ risk scores were computed as per the IRSS equation mentioned before (Supplementary Table S8), the KIRP samples were classified into low- and high-risk groups using the median IRSS value. We found that the risk score was negatively correlated with the OS rates (Figure 9A). In addition, the KM analysis (Figure 9B) indicated that the patients in the low-risk group had significantly better prognoses than those in the high-risk group (log-rank *p* = 0.049; HR = 1.84, 95% CI = 1.02–3.32. The model performed well in predicting OS as demonstrated by the AUCs of the survival ROC curves which were 0.601, 0.571, and 0.518 over 1, 3, and 5 years, respectively (Figure 9C). Several clinical features including age, gender, and pathologic stage of the tumor together with IRSS were utilized to construct a nomogram for predicting prognostic survival over 1, 3, and 5 years (Figure 9D).

Collectively, the IRSS that we have developed has potential as a prognostic predictive biomarker for both KIRC and KIRP and may be useful in other RCCs also.

## Discussion

In this study, we investigated the possibility of using immunity-related genes in developing a prognostic biomarker for RCCs. To do this, we used the TCGA-KIRC database as a training set. The differential gene analysis was performed to identify DEGs between the tumor and normal tissues in KIRC patients. Following this, GO and KEGG enrichment analyses were conducted on the DEGs to identify immune and tumor-related genes which were upregulated. Our results support earlier reports that IAGs are vital to the onset and advancement of KIRC (Shen et al., 2020).

Subsequently, we used the Cox proportional hazard model and LASSO regression analysis to select immunity-related genes from the DEGs identified through the previous analyses. We identified six independent immunity-related genes associated with KIRC prognosis. Data on these genes were integrated to create an IRRS model to predict patient prognosis for KIRC.

The progestagen-associated endometrial protein (PAEP), which is a member of the lipocalin family of glycoproteins, is one of the six genes identified in this study and functions as a negative controller of T cell receptor-mediated activation. In melanomas, tumor cells secrete PAEP which can inhibit the activation, proliferation, and cytotoxicity of T lymphocytes (Ren et al., 2015). The PI3-kinase/Akt signaling pathway is active in most RCCs. A recent study showed that dual inhibition of PI3K/mTOR by NVP-BEZ235 triggered growth arrest in RCC cell lines both *in vivo* and *in vitro* (Cho et al., 2010). SAA2 expression was positively linked to the disease stage of non-small cell lung cancer (Zhang et al., 2021). Another study has shown that SAA1 may be used as a biomarker for diagnosis and prognosis in advanced KIRC cases (Li et al., 2021). Many human malignancies exhibit dysregulated IL20RB expression, which is remarkably high in KIRC. Previous work has shown that IL20RB can be used as a prognostic biomarker for tracking KIRC treatment (Guo et al., 2022). Abnormal expression of IFI30 has also been found in many human tumors. For example, IFI30 overexpression is linked to the occurrence of high-grade tumors, immune infiltration, and worse OS in glioma (Jiang et al., 2021).

The KM analysis indicates that the IRSS model that we have developed is a robust biomarker for predicting patient prognosis in KIRC. The differences in OS between the high- and low-risk groups were statistically significant. Additionally, the survival ROC data showed that our model’s ability to predict prognosis was in line with real-world outcomes. Furthermore, survival analysis with clinicopathological data (such as age, histologic grade of tumor, pathologic stage of tumor, and gender) as covariates, showed that our IRSS model was still a reliable and independent prognostic predictor. We also developed a nomogram to predict patient survival using the IRSS and clinicopathologic data. The DCA results demonstrated that the IRSS that we have developed had greater accuracy in predicting patient prognosis than the conventional prediction using (tumor node metastasis, TNM) stage. Furthermore, we also found that the nomogram using clinical data along with the IRSS could make better predictions of patient prognosis for KIRC.

We have also investigated the relationships between the expression levels of six IAGs in KIRC and tumor infiltration by 24 different types of immune cells. Our results show that the expression levels of these six IAGvary substantially in the different infiltrating immune cells and that these expression levels were significantly and positively correlated with immune infiltration by Tregs. Immune homeostasis is influenced by Treg cells, which have high expression levels of the forkhead box P3 transcription factor (FOXP3) (Kasagi et al., 2014; Li et al., 2015; Ni et al., 2018). In the supplementary material, we also provide the results of an analysis that looks at the relationship between FOXP3 expression and the expression levels of the six IAGs in KIRC tissues. The results show a significantly positive association between these variables (Supplementary Figure S3). Similar results were found in KIRP tissues also (Supplementary Figure S4). Previous research has demonstrated that there are various mechanisms by which Treg cells can control the growth of tumors, including affecting the expression of immune checkpoint molecules (CTLA-4, ICOS, LAG-3) and secreting immunosuppressive cytokines (IL-10 and TGF-β) (Gu et al., 2022). Therefore, targeting these six IAGs may become an alternative strategy for tumor immunotherapy in treating KIRC.

The highly complex processes of tumor development and progression in RCCs are regulated by several genes. Currently, there are numerous relevant prognostic models for patients with KIRC as a result of an increase in interest in this area of research (Xu et al., 2020b; Wu et al., 2021). We used the LASSO regression analysis to establish a risk model linked to immunity-related genes for patients with KIRC. LASSO regression is a compressed estimation technique. By creating a penalty mechanism, constricting a few coefficients, and zeroing out some coefficients, it can produce a more refined model than univariate and multifactorial regression. It can also perform variable selection simultaneously with parameter estimation and is thus better at addressing the multicollinearity problems in regression analysis than univariate and multifactorial regression. The drawbacks, however, of this model are that it is difficult to calibrate when some coefficients are compressed, which results in underfitting of the data.

Our work has used the expression profiles of PAEP, PI3, SAA2, SAA1, IL20RB, and IFI30 to develop an IRSS model to predict the prognoses of patients with KIRC. In addition, we also generated 1-, 3- and 5-year ROC curves for patients with KIRC on the basis of the risk model; we find that our prognostic model is likely to offer a more thorough and individualized scheme of treatment. The IRSS that we have developed can not only predict clinical prognosis but can also reflect the immune status of patients with KIRC. However, the interaction among these six genes and their potential role in predicting prognosis, especially response to immunotherapy for KIRC, still needs to be investigated.

We also analyzed the mRNA expression levels of the six immunity-related genes identified in this study in KIRC and KIRP tissues. We find that the expression levels of all six immunity-related genes expression were higher in KIRP tumor tissues than in normal tissues (Supplementary Figure S5). However, only PAEP, SAA1, and IL20RB were overexpressed in KIRC tissues as compared to normal tissues (Supplementary Figure S6). These differences in expression need to be further verified. In addition, we investigated the protein levels of these six immunity-related genes in tumor tissues (KIRC/KIRP) and normal tissues using data from the human protein atlas (HPA) database (Supplementary Figure S7). We find that the protein levels and mRNA levels are not always consistent; this needs to be further investigated through immunohistochemistry studies.

## Conclusion

In summary, we have developed a new and reliable biomarker for predicting the prognosis of patients with KIRC using six immunity-related genes. The IRSS model developed was found to be quite robust and may be useful for clinical application. Our observations need to be verified with further research and additional experimental investigation.

## Data Availability

The datasets presented in this study can be found in online repositories. The names of the repository/repositories and accession number(s) can be found in the article/Supplementary Material.
