# Supplementary figures and images for: Construction and verification of a novel prognostic risk model for kidney renal clear cell carcinoma based on immunity-related genes
Source: Front Genet. 2023 Jan 20;14:1107294. doi: 10.3389/fgene.2023.1107294 (PMC9895858; doi:10.3389/fgene.2023.1107294)

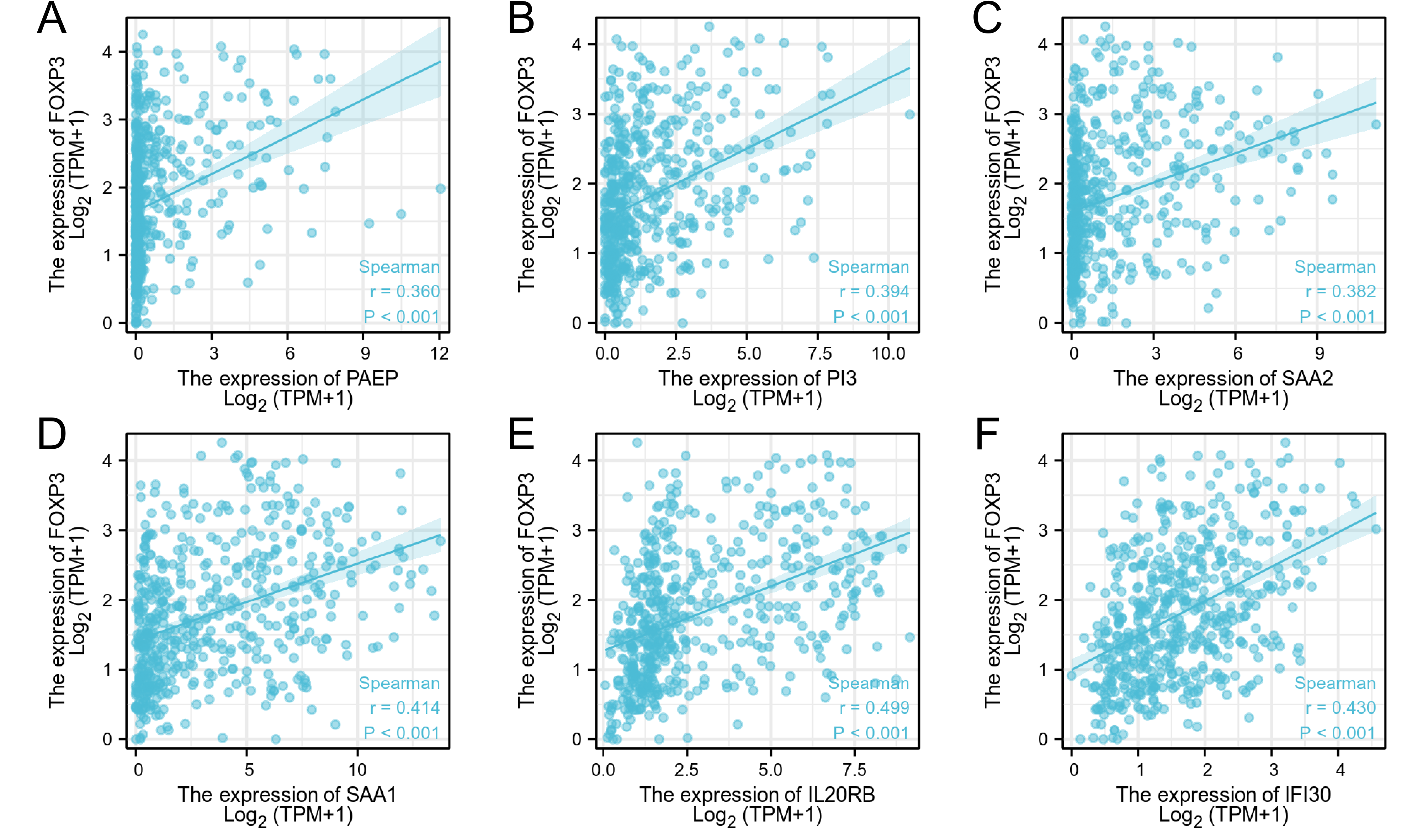

Supplement: Supplementary file 1 [file Image3.TIFF]

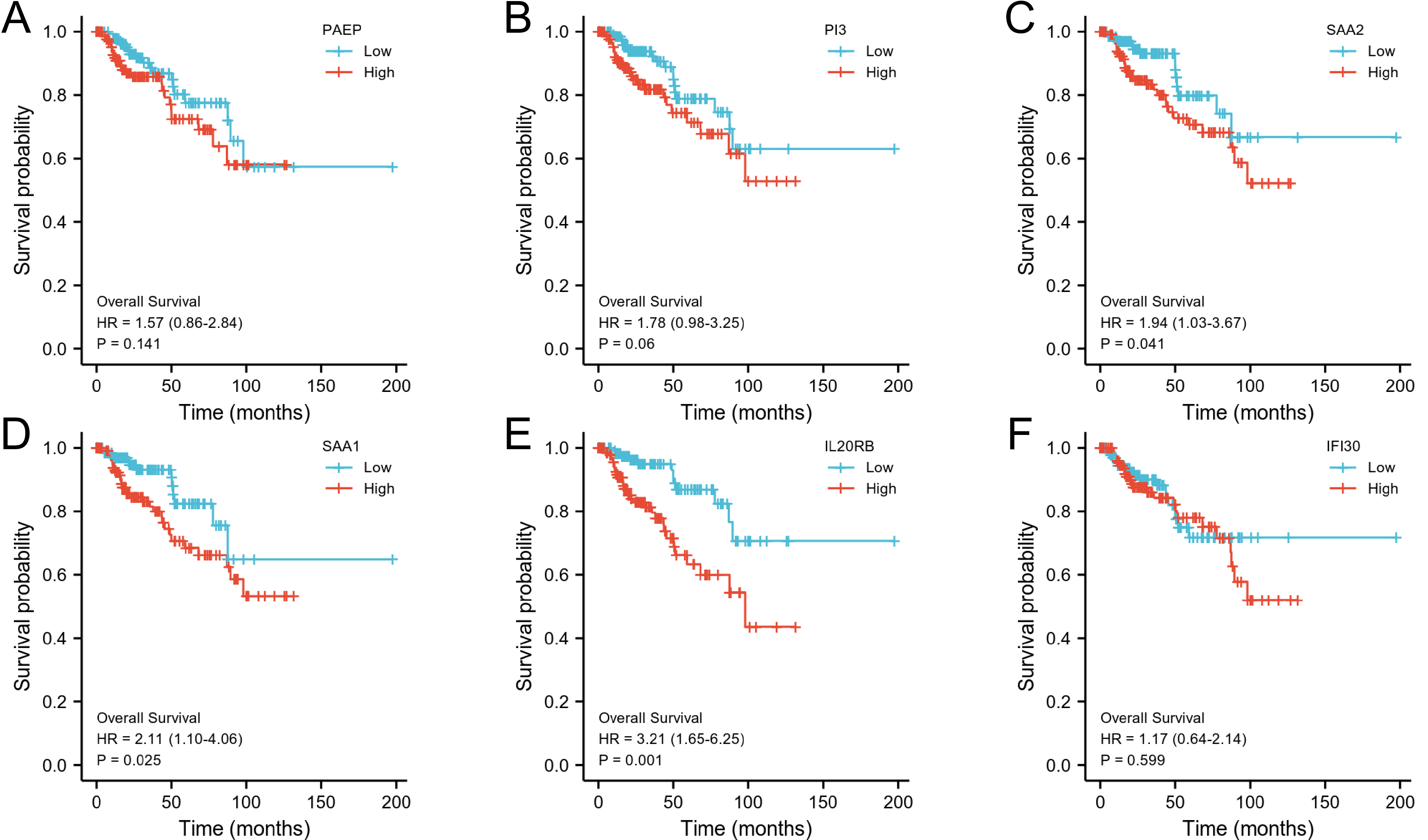

Supplement: Supplementary file 3 [file Image1.TIFF]

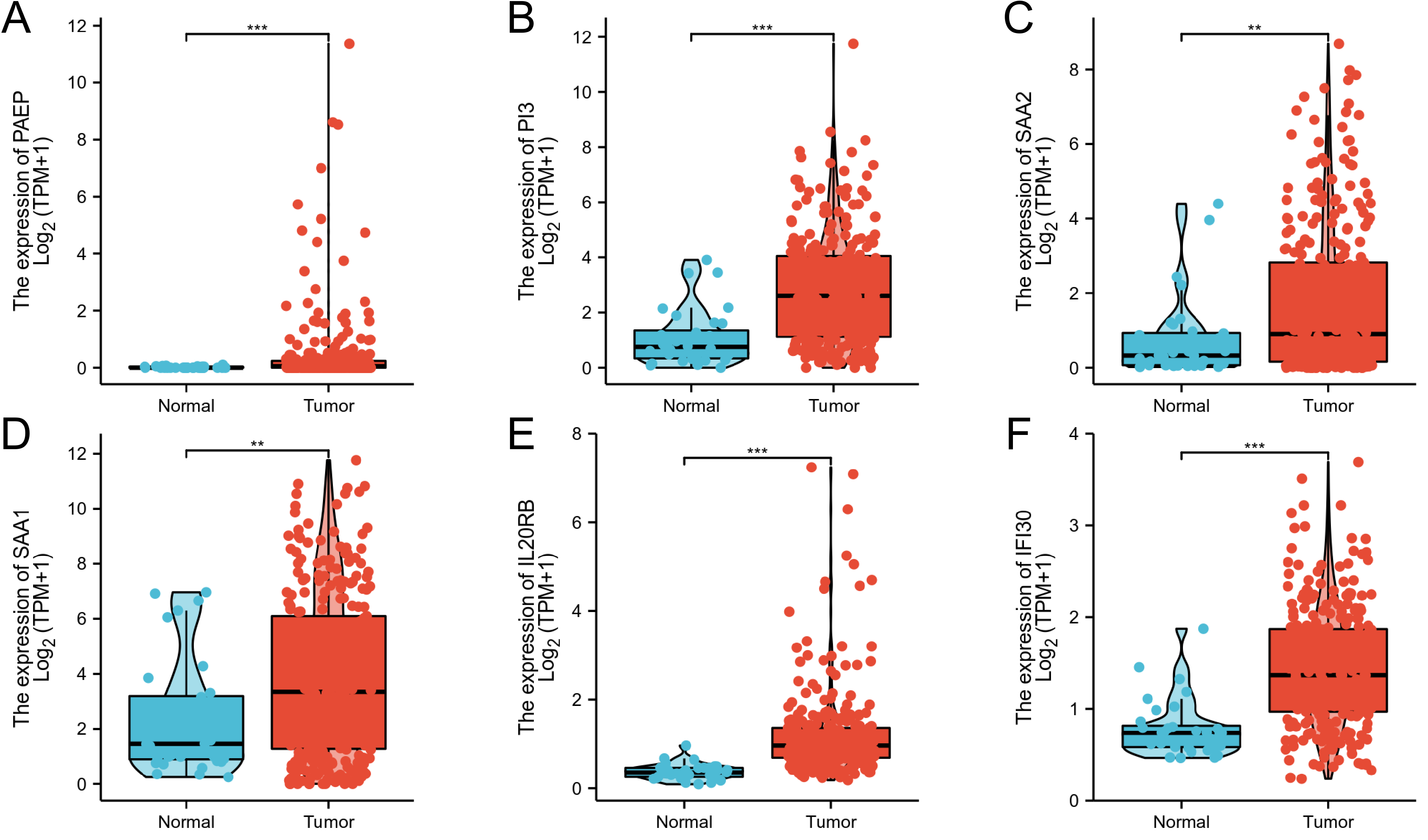

Supplement: Supplementary file 7 [file Image5.TIFF]

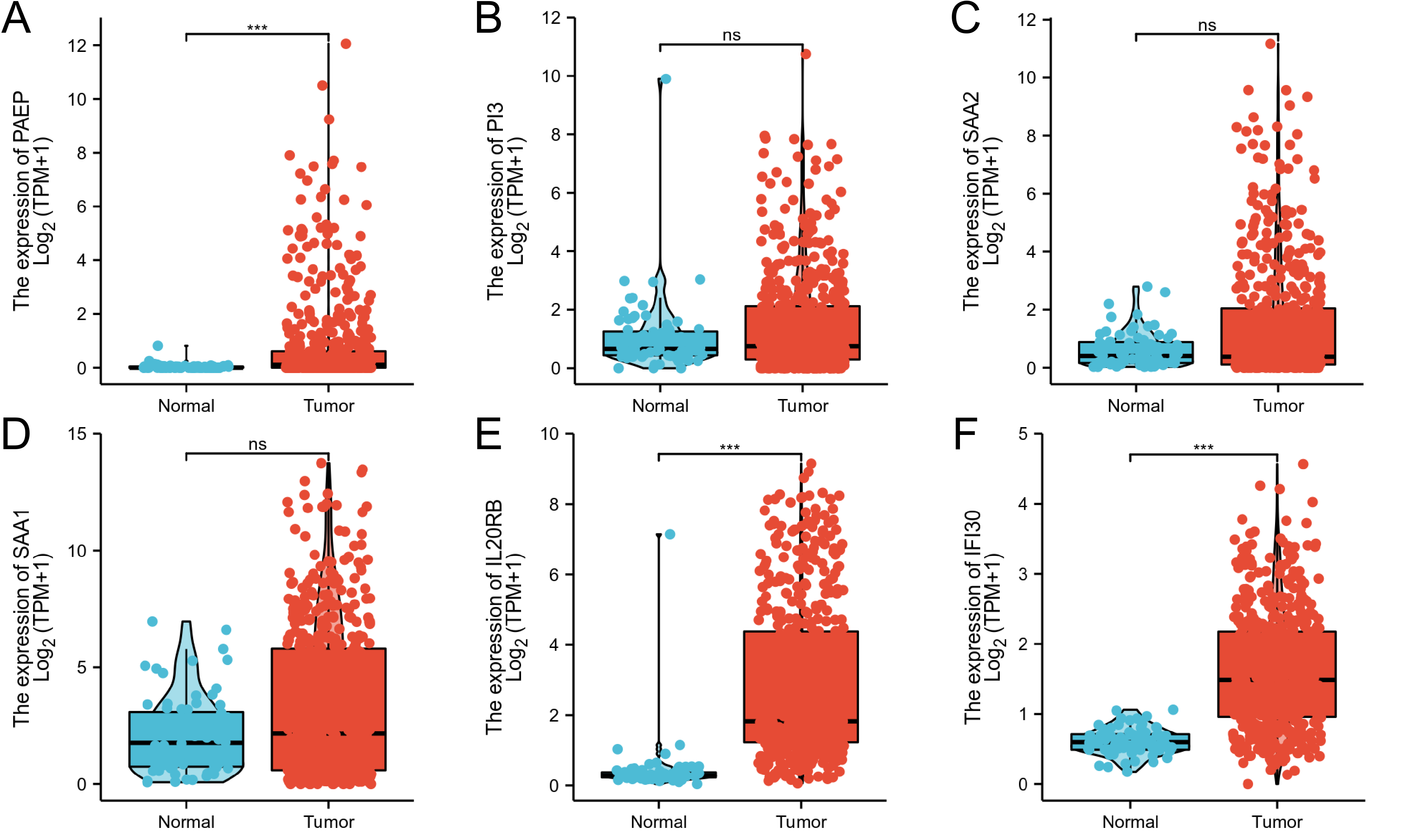

Supplement: Supplementary file 10 [file Image6.TIFF]

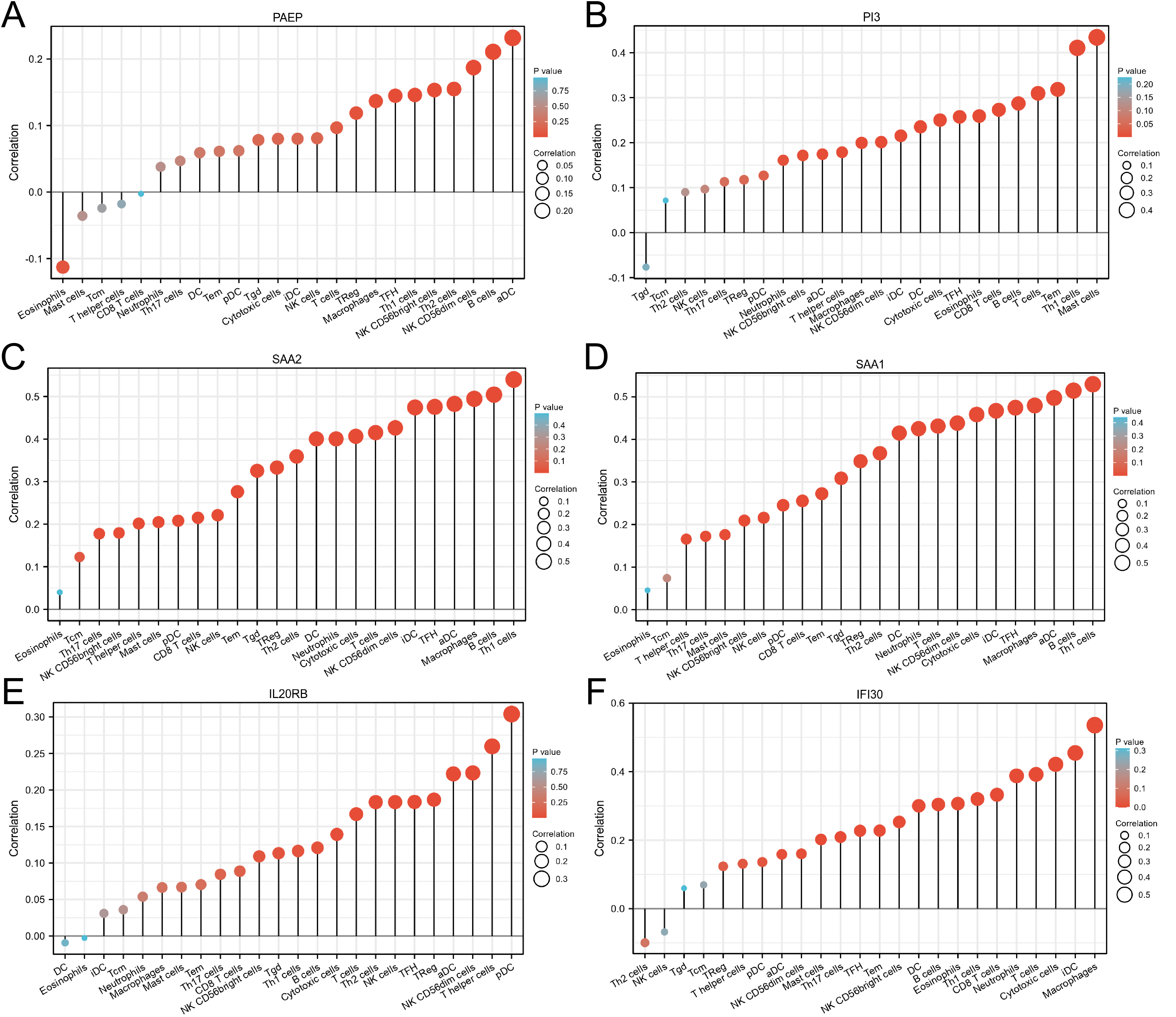

Supplement: Supplementary file 12 [file Image2.TIFF]

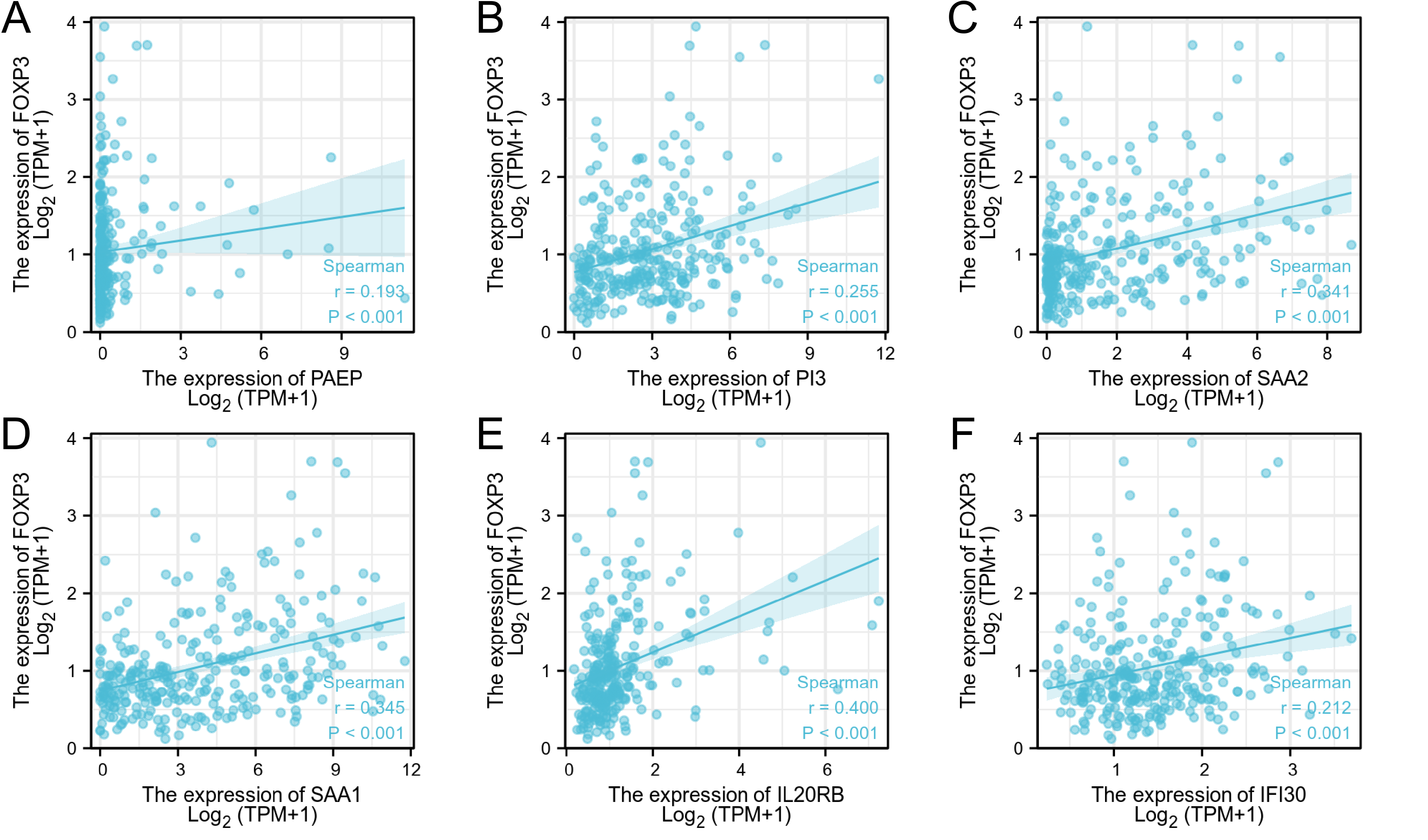

Supplement: Supplementary file 13 [file Image4.TIFF]

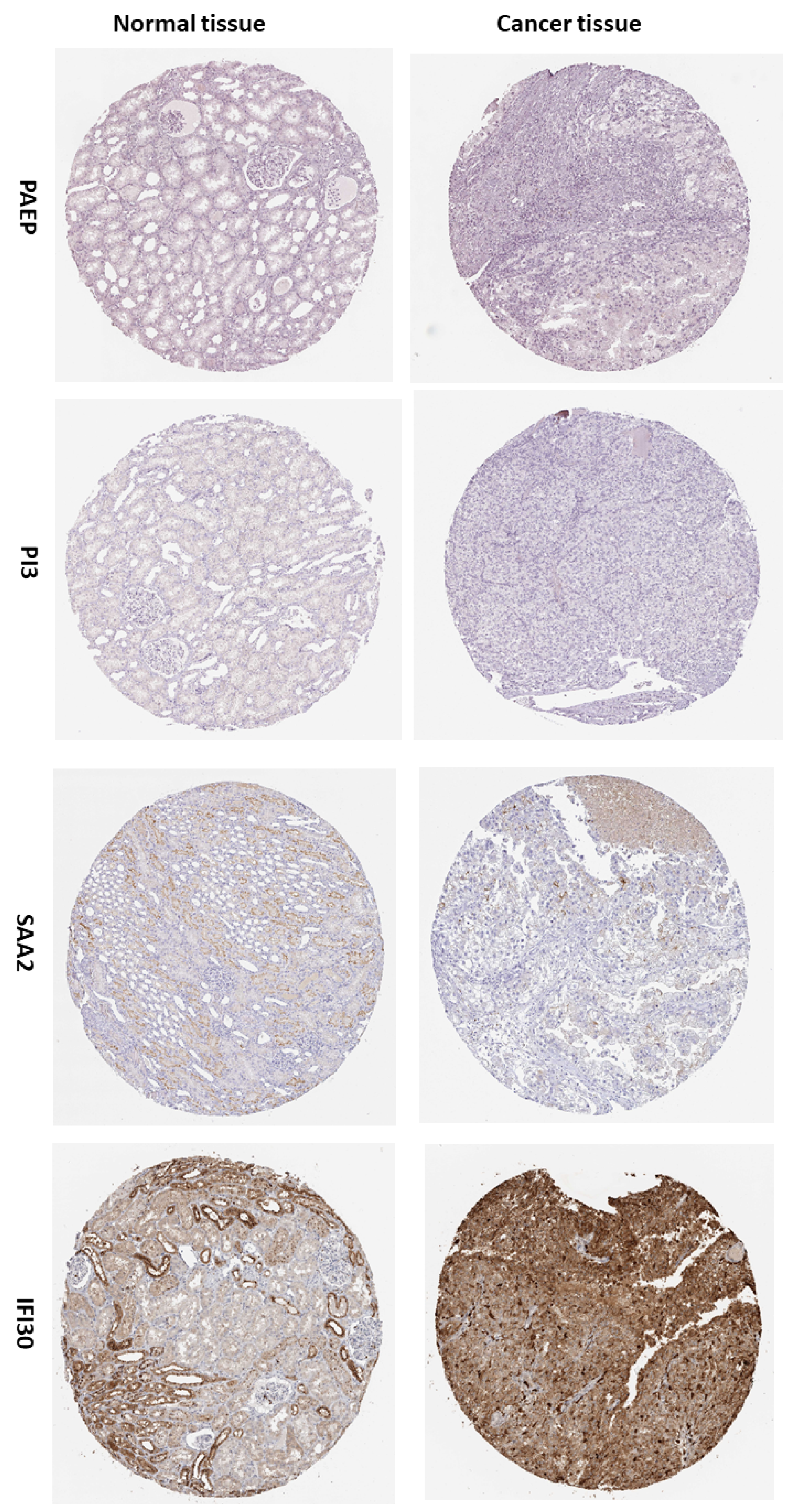

Supplement: Supplementary file 16 [file Image7.TIFF]
