# Supplementary material for: Construction and verification of a novel prognostic risk model for kidney renal clear cell carcinoma based on immunity-related genes
Source: Front Genet. 2023 Jan 20;14:1107294. doi: 10.3389/fgene.2023.1107294 (PMC9895858; doi:10.3389/fgene.2023.1107294)
Supplement: Supplementary file 5 [file Table1.DOCX]

| Characteristics | Total(N) | Univariate analysis | |  | Multivariate analysis | |
| --- | --- | --- | --- | --- | --- | --- |
|  |  | Hazard ratio (95% CI) | P value |  | Hazard ratio (95% CI) | P value |
| gender | 526 |  |  |  |  |  |
| MALE | 346 | Reference |  |  |  |  |
| FEMALE | 180 | 1.077 (0.788-1.471) | 0.643 |  |  |  |
| histologic_grade | 526 | 2.305 (1.880-2.826) | **<0.001** |  | 1.325 (1.044-1.682) | **0.021** |
| pathologic_stage | 526 | 1.879 (1.647-2.144) | **<0.001** |  | 1.584 (1.357-1.847) | **<0.001** |
| age | 526 | 1.032 (1.018-1.045) | **<0.001** |  | 1.034 (1.020-1.049) | **<0.001** |
| riskscore | 526 | 9.062 (5.330-15.409) | **<0.001** |  | 3.334 (1.785-6.229) | **<0.001** |
